# Tincture of Iodine in Small Pox

**Published:** 1854-02

**Authors:** J. Crawford

**Affiliations:** Prof. of Clinical Medicine in M’Gill College, Montreal


### *On the Application of Tincture of Iodine as an Ectrotic Remedy for Small-pox.*

BY J. CRAWFORD, M. D., PROF. OF CLINICAL MEDICINE IN M'GILL COLLEGE, MONTREAL.

It is now upwards of nine years since I first recommended the application of tincture of iodine as an ectrotic remedy for smallpox, and although I observe that the suggestion has been noticed by Dr. Copland, in his Dictionary of Medicine, and by Dr. Dunglison, in his work on Therapeutics, and also by some others in the United States, I would nevertheless desire again to draw the attention of the medical profession to the benefit that a more extended experience has convinced me would follow a general application of the remedy.

Epidemics of the dangerous malady of smallpox have been fortunately rare amongst us, and therefore the opportunity of further testing this remedy had not been afforded me, till the latter end of the last year, and earlier months of the present, during which period I have had occasion to treat, both in hospital and private practice several cases of very grave variolous disease, and would desire to lay my further experience before the profession, anxious that a fair trial and just estimate of the application should be made, while I feel fully confident that it will maintain the reputation I have deemed it deserving of. I would here appeal to those who have seen much of the natural smallpox, or its effects, how few cases escape pitting and unseemly scars, when the disease is allowed to run its course without interference, and I would only ask, how many attempts have been made in consequence, to supply an ectrotic remedy, and how difficult of application, or, disagreeable, and even inefficacious, are any that have been hitherto recommended. The Herculean undertaking of cauterizing the several individual pustules, in severe cases, quite precludes its application. I have reason to think the compound tincture of iodine a very powerful and efficacious remedy, which has been tried with very satisfactory results in the Montreal General Hospital by my friend Dr. Campbell, but from my being under the impression that the addition of the hydriodate of potass caused more pain, I have not employed this form. The disagreeable mercurial mask, the inefficacious covering of gold-leaf, cotton, or collodion, are now in a great measure laid aside. I stated formerly, on the occasion of my first suggestion of this application in the Medical Gazette published in this city in 1844, that I was led to try it in smallpox, from the very marked benefit I derived from its use in erysipelas, and various other cutaneous diseases, for several years previously. I was then satisfied of its *antiphlogistic* powers and *sootihng* effects, and trusted that a more general employment of it in variola would establish its claims to general confidence.

During the late epidemic of variola, I have had several opportunities of trying its powers, and my cases have been observed by many members of the profession, to whom the issue has afforded every satisfaction. I have reason also to know that several medical practitioners have followed my example with success, while others have made only a very *imperfect* and *insufficient occasional* application, which neither could afford a satisfactory result, nor determine the advantages derivable from it. I have been favored with the opinion of several physicians of this city, of the highest standing in the profession, on the advantage of using this remedy, which I subjoin. The application I have used is a saturated solution of iodine in spirits of wine, *which is to be brushed freely over the face once or twice daily, from the earliest day of the eruption that is practicable, and continuing the repetition of the application daily, or oftener, during the period of the maturation of the pustules, The earlier the application is commenced, the more efficacious it proves. The inflammatory and ulcerative processes are controlled, and the intolerable itching relieved,* by which means *scratching*, and its evil consequences, are obviated. For some time I was disposed to confine the application to the face, as being the part most disposed to ulceration and pitting, as well as that most desirable to be preserved from marks. I have, however, on many occasions applied it to various other parts, for the sake of experiment, or contrast. and also to relieve the intolerable pruritus, and have even extended it over nearly the whole body, at the patient's desire, without any evil consequences or inconvenience from the most extended application. The relief it afforded to the itching (if it conferred no other boon) would of itself be a sufficient recommendation of the application. Its antiphlogistic and febrifuge properities, however, are very manifest, and I have no doubt *modify* and *moderate the fever*, and thereby operate in a most salutary manner. The medical treatment I have combined with it is so simple and mild, that a great deal cannot be attributed to it; being merely small doses of calomel and Dover's powder occasionally, during the day and night, as a sedative. When pits are left, I have observed that they principally occur in the nose, and I am inclined to think that this may in some degree be owing to the insufficiency of the application to this sensitive part, or from the disagreable vapor causing irritation of the schneiderian membrane, or eyes, which makes the patient more desirous to escape from its application; but even this inconvenience may be easily obviated by keeping the eyes shut, and, if requisite, stopping the nostrils. The immediate effect of the the application is pain, which is more complained of by some than by others. It speedily subsides, and gradually abates in severity, after the first few applications; and the relief to the itching it affords is so gratifying to the patient, and the effects so manifest to the friends, that they always remark the contrast of the parts "painted" with those left "unpainted," and frequently request a further extension of the application.—*Montreal Med. Chron.*

